# Safety and Efficacy of Dotinurad on Uric Acid in Patients With Chronic Kidney Disease With Estimated Glomerular Filtration Rate Below 25 mL/Min/1.73 m²

**DOI:** 10.7759/cureus.57362

**Published:** 2024-03-31

**Authors:** Akira Mima, Hidemasa Gotoda, Shinji Lee

**Affiliations:** 1 Nephrology, Osaka Medical and Pharmaceutical University, Takatsuki, JPN

**Keywords:** proteinuria, estimate glomerular filtration rate, uric acid, chronic kidney disease, dotinurad

## Abstract

Introduction

Dotinurad is being developed as a selective uric acid reabsorption inhibitor. However, its effect on lowering serum uric acid (UA) levels in chronic kidney disease (CKD) patients with severe renal dysfunction is unknown. Therefore, the purpose of this study was to determine the effect of dotinurad on renal function in CKD patients with an estimated glomerular filtration rate (eGFR) below 25 mL/min/1.73 m^2^.

Methods

Seven patients with CKD who received dotinurad 0.5 mg to 4 mg per day were studied retrospectively. Changes in UA, eGFR, and urine protein-to-creatinine ratio (UPCR) were analyzed. The observation period was 10.9±2.1 months.

Results

Serum UA levels were decreased and maintained with dotinurad administration. Nevertheless, there were no improvements noted in renal function. Additionally, no serious adverse effects were identified in any of the patients throughout the observation period.

Conclusion

Although the sample size in this study was small, our findings demonstrate the efficacy of dotinurad in individuals with advanced CKD who have an eGFR lower than 25 mL/min/1.73 m^2^.

## Introduction

Hyperuricemia is defined as uric acid (UA) levels of 7.0 mg/dL or higher [1]. It may be caused by genetic mutations or polymorphisms that increase UA production or decrease its excretion. Hyperuricemia is thought to contribute to the development and exacerbation of chronic kidney disease (CKD) [2].

There are two possible mechanisms of renal injury due to hyperuricemia. One is a UA crystal-dependent mechanism; the UA crystals in the renal tubules and interstitium due to hyperuricemia are called UA nephropathy. The other is the UA non-crystal mechanism, where hyperuricemia itself may exacerbate CKD by increasing inflammation or oxidative stress [3].

Hyperuricemia is classified into three types: increased production of UA, decreased excretion of UA, or both increased production and reduced excretion (mixed type). Hyperuricemia occurs in about 60% of cases due to decreased excretion, 30% due to the mixed type, and the remaining cases are attributed to renal excess, with the majority being the decreased excretion type. This is believed to be partially attributed to the deterioration of CKD and a decline in glomerular filtration rate (GFR), resulting in elevated serum UA caused by a reduction in UA excretion [4]. Presently, medications aimed at reducing UA levels encompass xanthine oxidoreductase (XOR) inhibitors, which impede UA production, and uricosuric agents. Nevertheless, individuals with renal impairment are advised to use XOR inhibitors, irrespective of the type of hyperuricemia [5].

Uric acid excretion transporters include URAT1, which is present in the proximal tubule and is responsible for UA reabsorption, and ABCG2, OAT1, and OAT3, which are also involved in excretion [6,7]. Interestingly, these UA excretory transporters are involved in the renal excretion of both UA and the uremic substance, indoxyl sulfate. It is reported that uremic substances accumulate in the blood and various organs, not only increasing a risk factor for cardiovascular complications but also CKD [8]. Further, febuxostat, an XOR inhibitor, and the uricosuric agent benzbromarone could inhibit ABCG2-induced UA excretion [9]. These drugs were reported to increase uremic substances in rodents [10]. Therefore, in CKD patients with hyperuricemia, UA-lowering drugs that do not affect UA excretion transporters should be used.

Dotinurad, a new and selective uric acid reabsorption inhibitor (SURI), demonstrates potent and specific inhibition of URAT1 while exerting minimal impact on ABCG2, OAT1, and OAT3 [11]. However, its effect on lowering serum UA levels in chronic CKD patients with severe renal dysfunction, especially an estimated GFR (eGFR) below 25 mL/min/1.73 m^2^ is unknown.

## Materials and methods

Study design 

**Figure 1:**
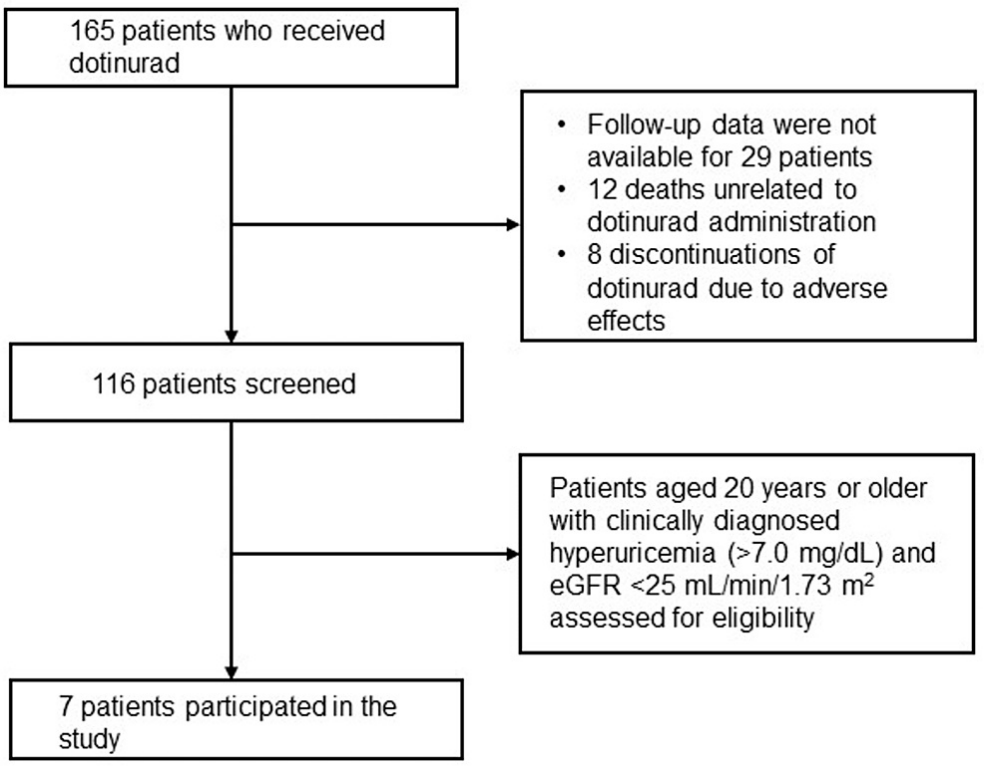
Flow diagram depicting the selection of eligible patients eGFR: Estimated glomerular filtration rate

This retrospective study examined and analyzed the medical records of patients who received outpatient care at the Department of Nephrology, Osaka Medical and Pharmaceutical University, Japan. Of the 165 patients who received dotinurad, follow-up data were not available for 29 patients. In addition, there were 12 deaths unrelated to dotinurad administration and eight discontinuations of dotinurad due to adverse effects (skin rash, liver dysfunction). Of the excluded patients, a collective of seven individuals aged 20 years or older were clinically diagnosed with hyperuricemia (>7.0mg/dL) and had an eGFR below 25 mL/min/1.73 m^2^, making them eligible participants. The procedures conducted with human participants in this study adhered to the ethical standards outlined by the National Research Committee and the 1964 Helsinki Declaration, along with its subsequent amendments or equivalent ethical norms. Informed consent was exempted, and an ethical review by the institutional review board was not conducted due to the final study's small sample size of seven patients (Figure 1).

Retrospective data collection or information from electronic medical records of Osaka Medical and Pharmaceutical University included data from biochemical analysis conducted using a Labospect 008 autoanalyzer (Hitachi Ltd., Tokyo, Japan). The extracted data encompassed details such as serum UA, triglycerides, total cholesterol, high-density lipoprotein (HDL) cholesterol, serum creatinine, urine protein-to-creatinine ratio (UPCR), and patient characteristics, including age, sex, medication, blood pressure, and BMI [12-14]. The eGFR for each patient was determined using the isotope dilution mass spectrometry (IDMS) traceable 4-variable Modification of Diet in Renal Disease (MDRD) formula, incorporating the 3-variable Japanese equation: 194 × serum creatinine - 1.094 × age - 0.287 × 0.739 (if female) [14-17].

Data analysis

Descriptive statistics (percentages and mean) were used to describe baseline characteristics and clinical characteristics of eligible patients. Data are reported as the mean±standard deviation. The Wilcoxon test was used for the analysis of variation in clinical data; p<0.05 was considered to indicate statistical significance. All analyses were performed using StatView (SAS Institute, Cary, CA, USA) and Excel software (Microsoft Corp., Redmond, WA, USA).

## Results

Table 1 captures the baseline characteristics of the seven patients (four males and three females). The average age at onset was 59.9±13.7 years. The observation period was 10.9±2.1 months. The average BMI was 21.8±5.1 kg/m^2^. All the patients in this study were treated with dotinurad (1.8±1.1 mg/day). Etiology of CKD were as follows: nephrosclerosis (n=3, 42.9%), antineutrophil cytoplasmic antibody-associated glomerulonephritis (n=2, 28.6%), diabetic kidney disease (n=2, 28.6%). Concomitant medications were as follows: febuxostat (n=1, 14.3%), sodium hydrogen carbonate (n=5, 71.4%), angiotensin receptor blockers (ARBs) (n=3, 42.9%), calcium channel blockers (n=4, 57.1%), alpha-blockers (n=1, 14.3%), alpha/beta blockers (n=1, 14.3%).

**Table 1:** Patient characteristics Total number of patients: n=7 CKD: Chronic kidney disease, ANCA: Antineutrophil cytoplasmic antibody, DKD: Diabetic kidney disease, ARBs: Angiotensin II receptor blockers

Characteristics	Values
Male:female ratio (n)	4:3
Age in years (mean±standard deviation)	59.9±13.7
BMI (kg/m^2^) (mean±standard deviation)	21.8±5.1
Observation period in months (mean±standard deviation)	10.9±2.1
Treatment dosage of dotinurad (mg/day) (mean±standard deviation)	1.8±1.1
Etiology of CKD	
Nephrosclerosis (n)	3
ANCA-associated glomerulonephritis (n)	2
DKD (n)	2
Concomitant drugs (n)	
Sodium hydrogen carbonate (n)	5
Anti-uric acid agents (n)	1
Febuxostat (n)	1
Anti-hypertensive agents (n)	7
ARBs (n)	3
Ca-blockers (nifedipine) (n)	4
α-blockers (doxazocin) (n)	1
αβ-blockers (carvedilol) (n)	1

Table 2 shows the baseline laboratory findings of the patients. Baseline total cholesterol was 147±21 mg/dL, total triglyceride was 91±19mg/dL, and HDL cholesterol was 55±12 mg/dL.

**Table 2:** Laboratory findings of the patients TC: Total cholesterol, HDL-C: High-density lipoprotein cholesterol, TG: Triglyceride, eGFR: Estimated glomerular filtration rate; UPCR: Urine protein-to-creatinine ratio, Cr: Creatinine

Characteristics	Results	Units	Reference range
Uric acid	8.6±1.9	mg/dL	Male: 3.6-7.0; Female: 2.3-7.0
TC	147±21	mg/dL	150-219
HDL-C	55±12	mg/dL	Male: 40-86; Female: 40-96
TG	91±19	mg/dL	50-149
eGFR	14.3±4.1	mL/min/1.73 m^2^	≥60
UPCR	3.3±2.6	g/gCr	<0.2

**Figure 2:**
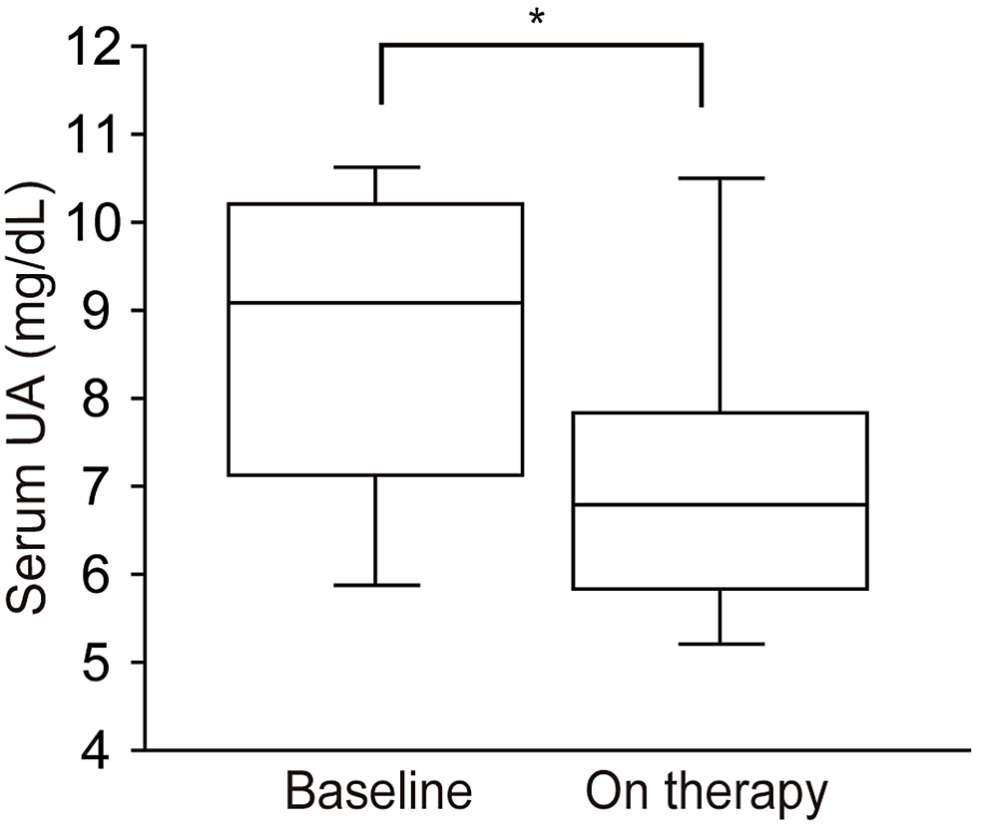
Box plots showing the levels of serum UA at baseline and during the dotinurad treatment ^*^p<0.05 UA: Uric acid

**Figure 3:**
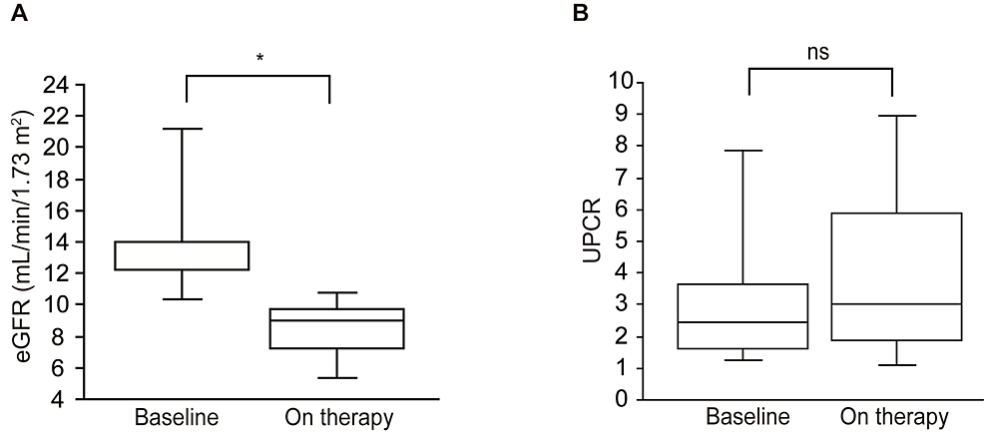
Change in eGFR (A) and UPCR (B) before and after the administration of dotinurad ^ *^p<0.05, ns: not significant eGFR: Estimated glomerular filtration rate, UPCR: Urine protein-to-creatinine ratio

Following the dotinurad treatment, there was a notable reduction in serum UA levels compared to the baseline (8.6±1.9 mg/dl at baseline vs. 7.1±2.0 mg/dl on therapy, p=0.028 (Figure 2 and Table 3). Concurrently, the eGFR exhibited a decrease (14.3±4.1 mL/min/1.73 m^2^ at baseline vs. 8.4±2.0 mL/min/1.73 m^2 ^on therapy, p=0.018, as depicted in Figure 3A and Table 3). Notably, the UPCR remained unchanged throughout the observation period (3.3±2.6 at baseline vs. 4.0±3.0 on therapy, p=0.499, illustrated in Figure 3B and Table 3).

**Table 3:** The p-values for each parameter in this study UA: Uric acid, eGFR: Estimated glomerular filtration rate, UPCR: Urine protein-to-creatinine ratio

Variable	p-value
UA	0.028
eGFR	0.018
UPCR	0.499

## Discussion

This study demonstrates, for the first time, the renal effects of dotinurad on serum UA in CKD patients with an eGFR below 25 mL/min/1.73 m^2^. Our results show that the induction of dotinurad treatment significantly decreased serum UA in those with advanced CKD. Though our study participants with CKD continued to have impaired renal function throughout the observation period, the results demonstrated that dotinuard, the UA excretory urate-lowering agent, could decrease serum UA levels under these conditions.

Annual changes in eGFR have been reported to be significantly improved in the dotinurad treatment group, but no improvement was seen in this study. It was suggested that the improvement in renal function with dotinurad was based on the finding that UA-lowering drugs inhibited the progression of glomerulosclerosis [18]. Another study showed that UA-lowering therapy may block the renin-angiotensin-aldosterone system and result in a reduction in glomerular hypertension. In contrast, the lack of urinary protein reduction in our results suggests that the effect of dotinurad on decreasing intraglomerular pressure is minimal. However, the dotinurad dose in our study was relatively low, so studies at higher doses are needed [19].

As CKD progresses, UA excretion via ABCG2 in the intestine increases. As ABCG2 is also involved in the excretion of uremic toxins, such as indoxyl sulfate, the disruption of intestinal flora in CKD may further exacerbate CKD by increasing uremic toxins [9,10]. Conversely, we have shown that normalization of the intestinal flora by prebiotics inhibits the progression of CKD [20]. In addition, the position of sodium-glucose cotransporter-2 (SGLT2) inhibitors in the treatment of CKD is important [21-23]. Interestingly, SGLT2 inhibitors increase the transcriptional activity of the ABCG2 gene via phosphorylation of cAMP response element binding [24].

The liver is the main site of UA synthesis, and about 70% of it is eliminated through the kidneys, while the remaining 30% is excreted by the small intestine [25]. Especially, the expulsion of UA from the ileum is facilitated by ABCG2. Therefore, it is likely that dotinurad affects ABCG2-induced UA excretion a little and may aid the excretion of UA from the kidney and small intestine [6,7]. This could be associated with the remarkably high success rates in reaching serum UA levels below 6 mg/dL, with 91.3% achieved at a dosage of 2 mg/day and 100% at 4 mg/day, as noted in a 58-week open-label phase 3 study of dotinurad [26]. Furthermore, our previous study also showed the administration of dotinurad effectively decreased serum UA levels, with an achievement rate of 76% for a UA level in the range of 6 mg/dL in patients with CKD [27].

In diabetic kidney disease (DKD), diabetic conditions activate protein kinase C (PKC), modulating cellular signaling in podocytes and glomerular endothelial cells [28,29]. This activation can affect inflammatory cytokines, including nuclear factor-kB, tumor necrosis factor-α, and interleukin-6 [30]. Further, PKC activation in glomeruli is linked to transforming growth factor-β signaling, increasing collagen type IV, and resulting in mesangial expansion [31]. Increased levels of UA have been reported to increase inflammation and oxidative stress, leading to the accumulation of extracellular matrix [32]. Therefore, strict UA control is necessary to inhibit the progression of CKD.

Our study may be limited by the small number of patients and the retrospective nature of the analysis performed on a small cohort of patients at a single institution. Although this study demonstrates the efficacy and safety of dotinurad in patients with CKD, further studies are needed.

## Conclusions

This retrospective study explored the impact of dotinurad on individuals with CKD whose eGFR was below 25. The findings indicate that dotinurad successfully and safely decreases UA levels. Nevertheless, it did not influence the decline rate of eGFR or UPCR. Interestingly, dotinurad demonstrated efficacy in reducing serum UA levels in CKD patients experiencing ongoing renal function decline.
